# Microscopic study of zinc nanoparticles synthesised using thermosetting polymer

**DOI:** 10.1186/s42649-019-0018-0

**Published:** 2019-12-31

**Authors:** Giriraj Tailor, Jyoti Chaudhay, Deepshikha Verma, Bhupendra Kr. Sarma

**Affiliations:** 1 Department of Polymer Science, M.L.S. University, Udaipur, Rajasthan 313001 India; 2 Department of Chemistry, Mewar University, Chittorgarh, Rajasthan 312901 India

**Keywords:** SEM, TEM, Zinc and XRD

## Abstract

The present study reports the novel synthesis of Zinc nanoparticles (Zn NPs) by thermal decomposition method and its characterisation by Scanning Electron Microscope (SEM), Transmission Electron Microscope (TEM), and X-ray Diffraction Measurements (XRD). Synthesis of Zn NPs was achieved by using thermosetting polymer and zinc salts as precursor. Zn NPs were obtained on calcination at 850 °C for 30 min. SEM study reveals that synthesized nanoparticles are spherical in shape. XRD analysis shows that the Zn NPs formed are low crystalline in nature.

## Introduction

Nanoparticles (NPs) have unique properties because of small size and higher surface to volume area. These particles are being increasingly used in many fields. Research interest on nanoparticles and its size-dependent properties has increased in last three decades (Templeton et al. 2000; Bönnemann et al. 2001; El-Sayed 2001). Zinc nanoparticles are being widely used in a variety of fields due to their useful electrical, optical, dermatological and antibacterial properties (Tomaszewska-Grzedaa et al. 2005; Kamaldeep and Dubey 2012; Rajeevan 2014). Zinc oxide nanoparticles have a band gapof 3.37 eV, which is relevant for various applications (Naif Abdullah and Mariadhas 2018). Further interest on zinc nanoparticles has increased as removal of the element zinc from dyes and water pollutants of textile effluents have been achieved (Xiaoxia et al. 2012; Pieqiang and Guohua 2012). Zn NPs may be used as effective control tools against mosquito larval populations and have potential applications in the pharmaceutical and biomedical field (Naif Abdullah and Mariadhas 2018).

Because of tremendous application possibilities, the chemical literature is replete with different approaches to the synthesis of Zn NPs. Preparation by chemical methods (Pieqiang and Guohua 2012; Sheree et al. 2007; Surabhi et al. 2013, Ayodele 2018) like sol-gel processing, precipitation, electro-deposition and thermal methods has been reported. However, the thermal decomposition method is widely used over other methods because of its many advantages (Ayodele 2018; Tailor et al. 2018). The cost of production in this method is minimal due to simpler equipment and cheaper chemicals. Moreover, more environment-friendly and less hazardous materials are utilised and stable monodispersed products may be obtained. Zinc salt precursors in the nano synthesis of these particles have been reported to play effective role on the surface morphology and properties (Rajeevan 2014). So, it is important to characterise the shape, size and location of the synthesised Zn NPs by microscopy methods. In this paper, we report the synthesis of zinc nanoparticles by thermal decomposition method and its characterisation by X-ray diffraction, Scanning Electron Microscopy (SEM) and Transmission Electron Microscopy (TEM). The objective of the study was to characterise the Zn NPs obtained by thermal decomposition of the Zn-polymer complex prepared in the laboratory through different microscopy methods in order to validate the usefulness of the cost-effective method for the synthesis of potential high-utility Zn NPs.

## Materials and methods

### Synthesis of the nanoparticles

All chemicals used in the experiment are of analytical grade. Phenol (C_6_H_5_OH), Formaldehyde (HCHO), Zinc Chloride (ZnCl_2_), Hydrochloric Acid (HCl) and Acetic acid (CH_3_COOH) were purchased from Central Drug House Ltd. India. All chemicals were used as received from the supplier. Deionised water was used throughout the experiment.

The synthesis was carried out in two steps:

- Step 1: Synthesis of polymer metal complex
- Step 2: Synthesis of zinc nanoparticle

**Step 1:**

Three round-bottomed necked flasks equipped with stirrer and reflux condensers were charged with 1 N metal salt solution, phenol and formaldehyde. The mixture was agitated thoroughly and was allowed to cool off. The drawn off and the remaining water was removed by slowly raising the temperature to 50 °C and applying vacuum by means of water pump. This temperature was maintained until sample from the melt, which solidified on cooling forming a pink solid substance, is separated out. The reaction was exothermic in nature. The dried solid sample was purified by washing with distilled water. The excess metal ion and impurities on the sample were removed on washing with water.

**Step 2:**

The synthesis of zinc nanoparticle was done by thermal decomposition method. The polymer metal composite was allowed to decompose at 850 °C for 30 min. Black fine particles were obtained.

### Purification of zinc nanoparticles

The synthesised nanoparticles were purified in the following steps:

- Step-1: Removal of volatile Impurity: At the time of decomposition many volatile impurities got separated.
- Step-2^:^ Removal of Metallic Impurities: Metallic ions were removed from the nanoparticles by keeping it in 12 N hydrochloric acid solution for 24 h. The mixture was then centrifuged and washed with distilled water till hydrochloric acid was completely removed (Chaudhary et al. 2017).

The chemical reactions of the synthesis process are given below:

### Characterization of the zinc nanoparticles

#### Scanning Electron Microscopy

Samples were investigated by Nova Nano FE-SEM 450 (FEI) Scanning Electron Microscope (SEM) to obtain topological, morphological and compositional information. Lens mounted DBS and LVD offer best selection of information and image optimization. Beam landing energy cam go down from 30 KeV to 50ev and resolution of 1.4 nm at 1 kV (TLD-SE) and 1 nm at 15 kV (TLD-SE). The entire sample was coated with gold before SEM analysis. Energy Dispersive X-ray spectroscopy (EDS) were also recorded in the SEM analysis.

#### Transmission Electron microscopy

Transmission electron microscopy (TEM) was performed for characterizing size and shape of synthesized zinc nanoparticles. It was performed on a Tecnai G_2_ 20 (FEI) S- Twin electron microscope at accelerating voltage of 20 kV. Specimens for TEM measurement were prepared by depositing a drop of colloid solution on a 400 mesh copper grid coated by an amorphous carbon film and evaporating the solvent in air at room temperature.

#### X- ray diffraction (XRD) analysis

XRD patterns were recorded on Philips PW 3050/10 model. The sample was recorded on a Philips X-Pert MMP diffractometer. The diffractometer was controlled and operated by a PC computer with the programs P Rofit and used a MoK (source with wavelength0.70930 A°, operating with Mo-tube radiation at 50 kV and 40 mA.

## Results and discussion

**Fig. 1:**
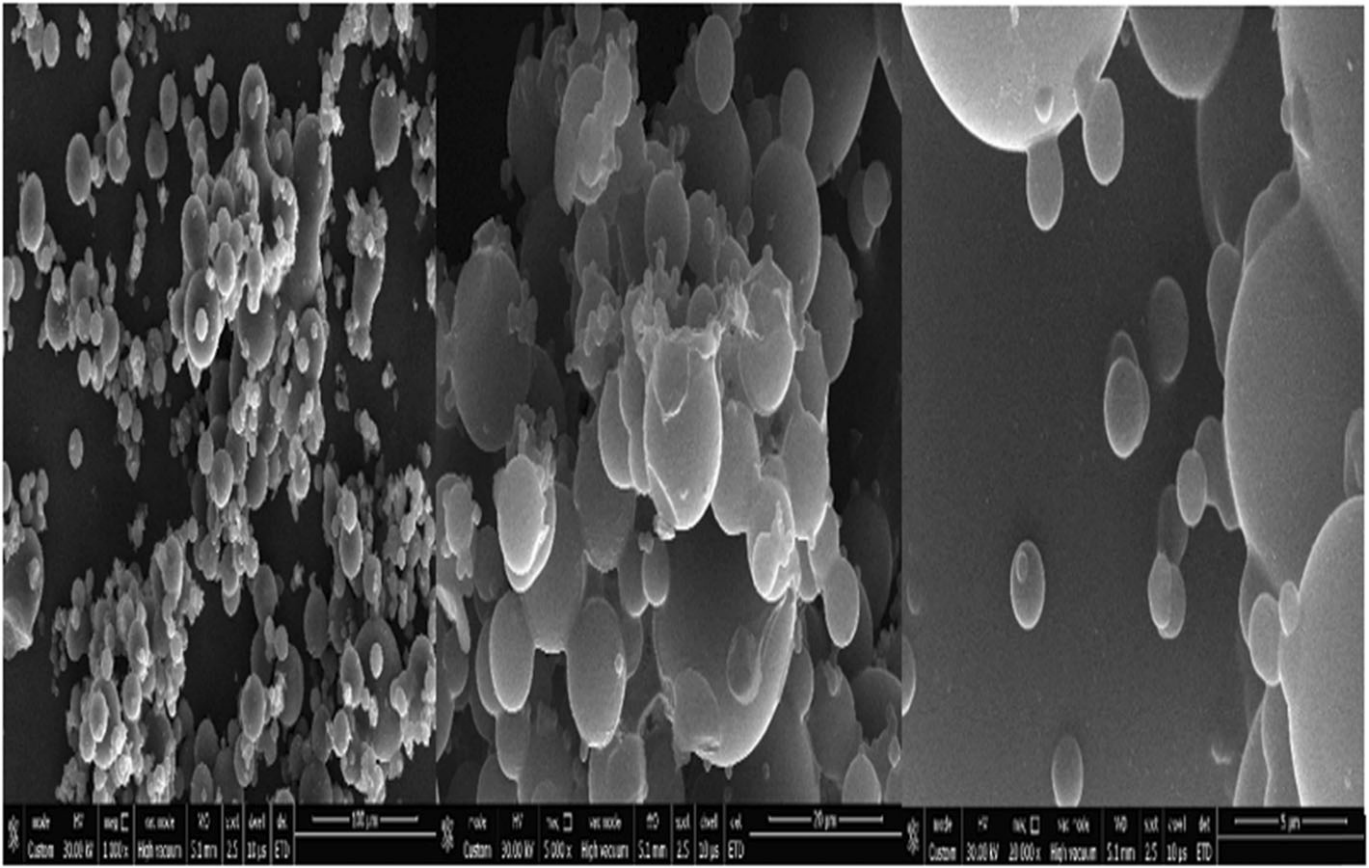
SEM images of Zinc Nanoparticles (**a**) 1000X Mag. **b** 5000X Mag (**c**) 20,000X Mag

**Fig. 2:**
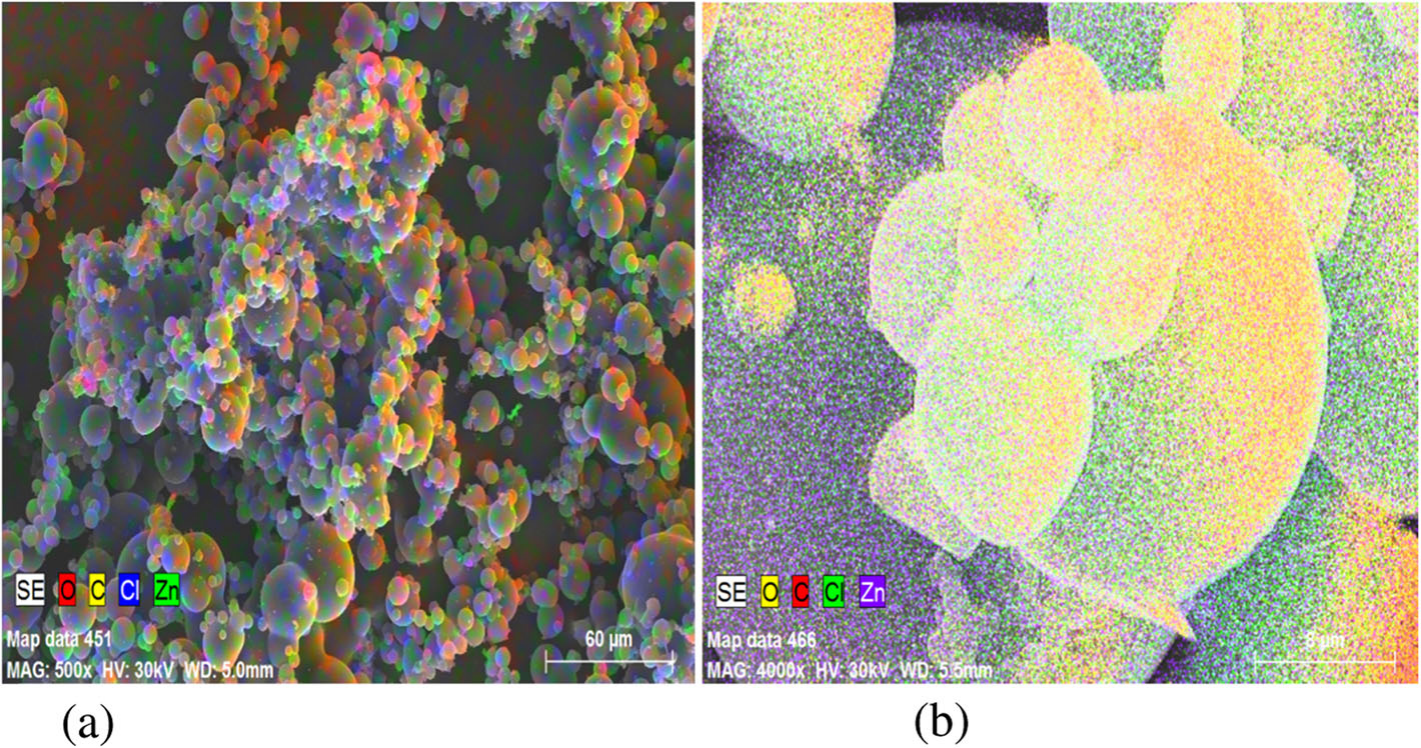
Energy Dispersion X-ray spectroscopy images of the NPs (**a**) 500 X Mag (**b**) 4000XMag

**Fig. 3:**
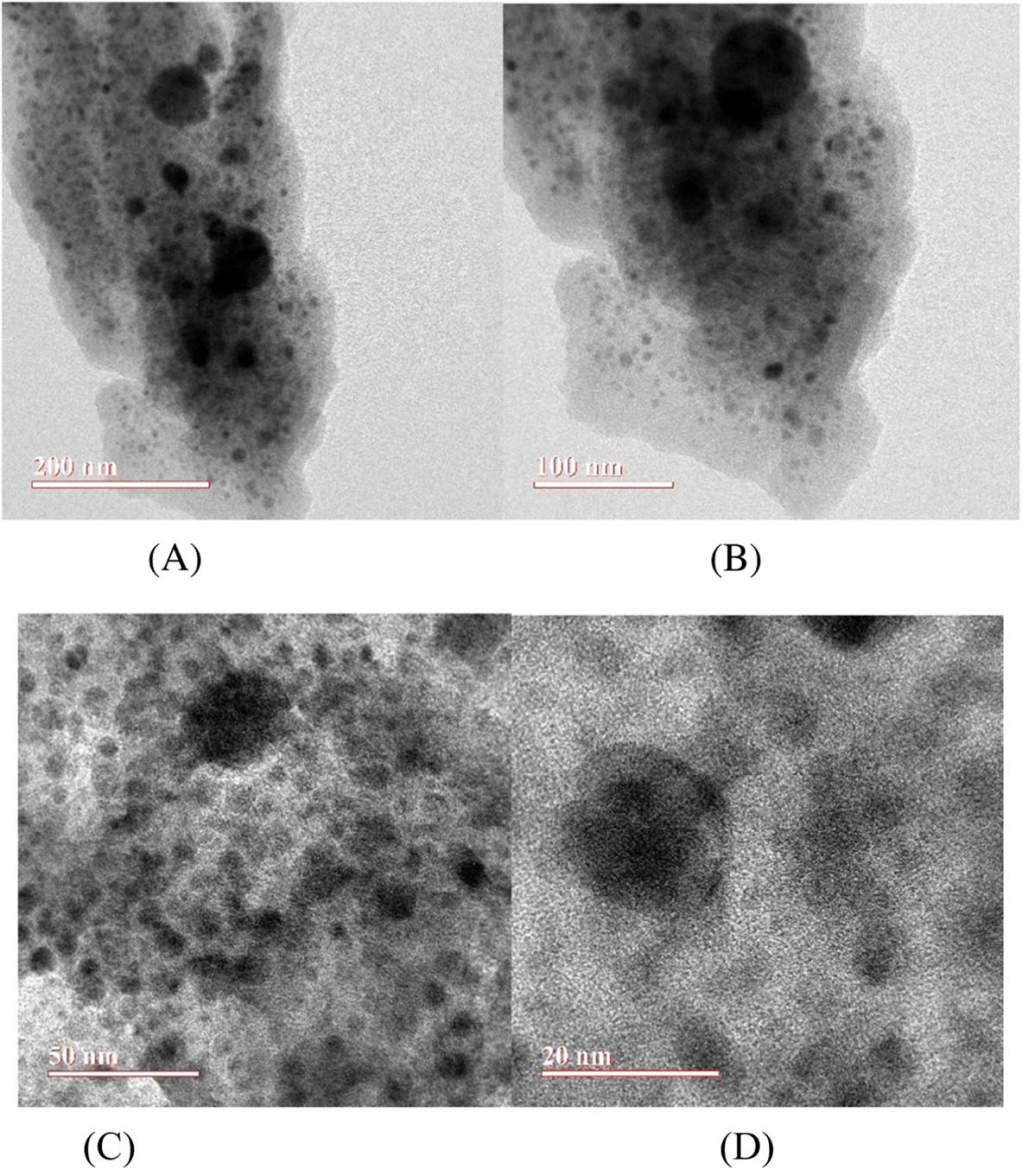
TEM images of Zinc Nanoparticles at different scale. **a** 200 nm (**b**) 100 nm (**c**) 50 nm (**d**) 20 nm

SEM image of the synthesised Zn NPs of the present study is shown in Fig. 1. The SEM image shows agglomerations of individual zinc nanoparticles. A closer look at the agglomerated lump shows the presence of several nanoparticle aggregates. In Fig. 1(c), particles appear to be agglomerated and some individual crystals are visible. Figure 2 represents the EDS images of the Zn NPs. These images contain the elemental maps of C, O, Cl and Zn and provide chemical information about the NPs synthesised. Fig. 3 represents the TEM photographs of zinc nanoparticles and these clearly show that spherical zinc nanoparticles has been obtained by the chemical method (Joghee et al. 2018). Although these particles overlap each other, the overall dispersion effect is good. The maximum size of the nanoparticles measured in TEM analysis is 59.57 nm.

**Fig. 4:**
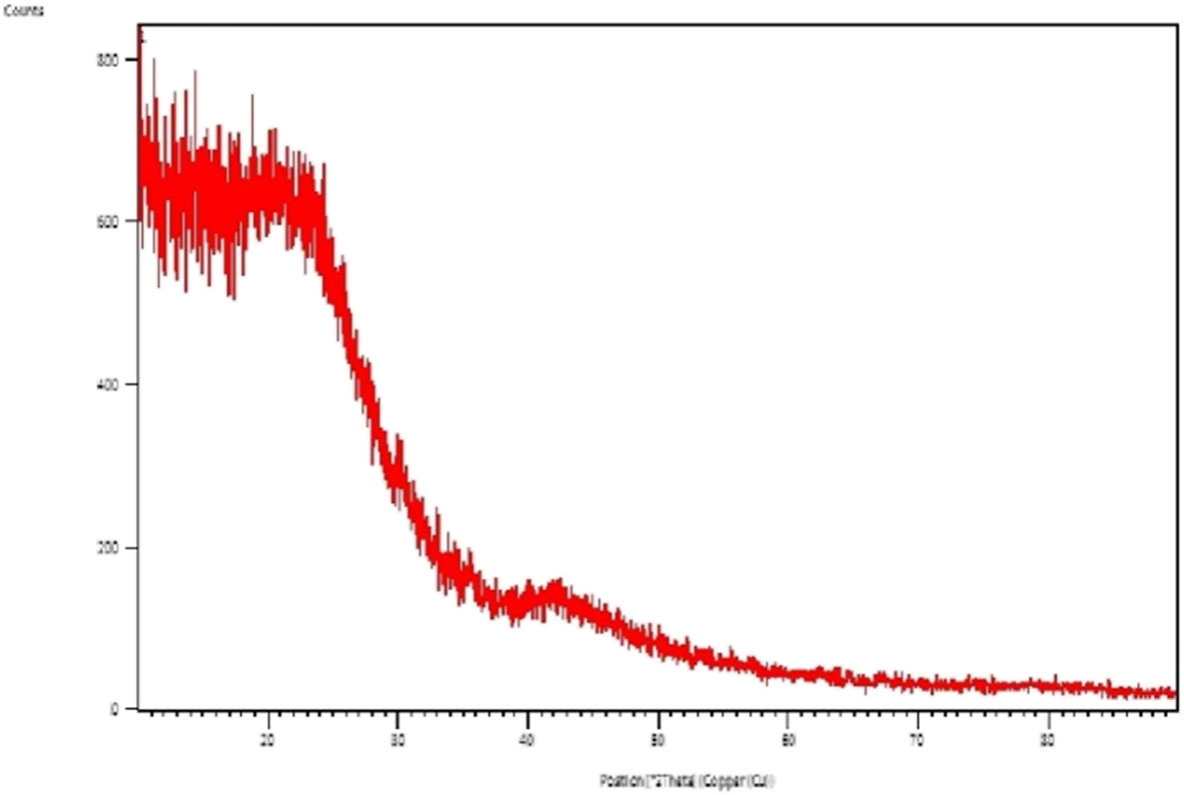
XRD spectra of zinc nanoparticles

Figure 4 shows the X-ray diffraction of zinc nanoparticles. XRD analysis, however, does not conclusively indicate the crystalline nature of the Zn NPs. XRD is a bulk analysis technique. The low crystalline nature of the Zn NPs could be attributed to the angle strain between monomers. Extended hexagonal network formation is geometrically unfavourable.

## Conclusion

Synthesis of zinc nanoparticles has been achieved by a simple and cheap chemical method. The synthesis was carried out via precipitation of Zn-polymer complex at 50 °C temperature followed by calcination at 850 °C. The successful formation of zinc nanoparticles has been confirmed by XRD, EDS, SEM, and TEM. The spherical structure of zinc nanoparticles was confirmed by SEM and TEM. XRD results confirm that the nanoparticles formed are low crystalline in nature because angle strain between the monomers. Our results conclusively prove that the cost-effective and less-polluting thermal decomposition method may be effectively employed to synthesise zinc nanoparticle which can have further industrial applications.

## Data Availability

Not applicable. “Please contact with author for data request”.

## Abbreviations

2D: Two Dimensional
3D: Three Dimensional
EDS: Energy Dispersion X-ray Spectroscop
NPs: Nanoparticles
SEM: Scanning Electron Microscopy
TEM: Transmission Electron Microscopy
XRD: X-Ray Diffraction
Zn NPs: Zinc Nanoparticles
