# Publisher Correction to: Applied Microscopy

**DOI:** 10.1186/s42649-020-0025-1

**Published:** 2020-02-05

**Authors:** Applied Microscopy

**Affiliations:** London, UK

An error occurred during the publication of the below article in *Applied Microscopy*. It was published in volume 50 with incorrect citation number.

In this correction article the old and new citation metadata is published in Table 1.

**Table 1:** Overview of incorrect and correct citation metadata

DOI	Incorrect volume number	Correct volume number	Incorrect citation number	Correct citation number
10.1186/s42649-019-0018-0	50	49	1	20

The original article has been updated. The publisher apologizes for the inconvenience caused to our authors and readers.

